# The Impact of Competition and Allelopathy on the Trade-Off between Plant Defense and Growth in Two Contrasting Tree Species

**DOI:** 10.3389/fpls.2016.00594

**Published:** 2016-05-04

**Authors:** Catherine Fernandez, Yogan Monnier, Mathieu Santonja, Christiane Gallet, Leslie A. Weston, Bernard Prévosto, Amélie Saunier, Virginie Baldy, Anne Bousquet-Mélou

**Affiliations:** ^1^Institut Méditerranéen de Biodiversité et d'Ecologie Marine et Continentale - Aix Marseille Université - Centre National de la Recherche Scientifique - IRD - Avignon UniversitéMarseille, France; ^2^Laboratoire d'Ecologie Alpine - Université de Savoie-Mont-BlancChambéry, France; ^3^Graham Centre for Agricultural Innovation- Charles Sturt UniversityWagga Wagga, NSW, Australia; ^4^Institut National de Recherche en Sciences et Technologies Pour l'Environnement et l'AgricultureAix-en-Provence, France

**Keywords:** allelopathy, competition, ecometabolomics, metabolic profiling, phenotypic response, *Pinus halepensis*, *Quercus pubescens*, secondary metabolism

## Abstract

In contrast to plant-animal interactions, the conceptual framework regarding the impact of secondary metabolites in mediating plant-plant interference is currently less well defined. Here, we address hypotheses about the role of chemically-mediated plant-plant interference (i.e., allelopathy) as a driver of Mediterranean forest dynamics. Growth and defense abilities of a pioneer (*Pinus halepensis*) and a late-successional (*Quercus pubescens*) Mediterranean forest species were evaluated under three different plant interference conditions: (i) allelopathy simulated by application of aqueous needle extracts of *Pinus*, (ii) resource competition created by the physical presence of a neighboring species (*Pinus* or *Quercus*), and (iii) a combination of both allelopathy and competition. After 24 months of experimentation in simulated field conditions, *Quercus* was more affected by plant interference treatments than was *Pinus*, and a hierarchical response to biotic interference (allelopathy < competition < allelopathy + competition) was observed in terms of relative impact on growth and plant defense. Both species modulated their respective metabolic profiles according to plant interference treatment and thus their inherent chemical defense status, resulting in a physiological trade-off between plant growth and production of defense metabolites. For *Quercus*, an increase in secondary metabolite production and a decrease in plant growth were observed in all treatments. In contrast, this trade-off in *Pinus* was only observed in competition and allelopathy + competition treatments. Although *Pinus* and *Quercus* expressed differential responses when subjected to a single interference condition, either allelopathy or competition, species responses were similar or positively correlated when strong interference conditions (allelopathy + competition) were imposed.

## Introduction

Interference between plants typically refers to either competition for resources (e.g., nutrients, light, water) or chemically-mediated interference (i.e., allelopathy) (Reigosa et al., 1999; Schenk, 2006; San Emeterio et al., 2007). Traditionally, resource competition has been regarded as the most important driver of plant community diversity and dynamics (Tilman, 1982; Schluter, 2000). However, recent research has shown that allelopathy can also affect the patterning of plant communities (Callaway and Ridenour, 2004; Fernandez et al., 2013). In this process, phytochemicals released into the environment inhibited the germination and growth of neighboring plants by altering their metabolism or impacting their soil community mutualists. Most of these studies have focused on plant invasion and the Novel Weapons Hypothesis (NWH). According to the NWH, allelopathic effects are purported to be strongest on species lacking historic exposure to the particular allelochemicals (Callaway and Aschehoug, 2000; Bais et al., 2004). A limited conceptual framework exists for the role of plant chemicals in the natural dynamics of co-evolved native species (Inderjit et al., 2011; Meiners, 2014), but it has been suggested that allelopathic interference may prove to be as important as competition for resources in modulating plant community function and dynamics. Therefore, it is crucial to evaluate the relative importance of these two plant interference mechanisms [resource competition (C) and allelopathy (A)] in experimentation, even if it is difficult and often unrealistic to separate these interactions in complex ecosystems.

Plants are thought to perceive their surrounding environment by using information on the distribution of essential resources (light, nutrients, and water) or chemical cues (volatile compounds, root exudates, leachates; Novoplansky, 2009; Weston and Mathesius, 2013). In response to interference, plants display a multitude of plastic responses to optimize their performances upon exposure to biotic stress (Pierik et al., 2013) and species differ in the way they are impacted by neighboring plants. Plants exhibit altered competitive and defense abilities in response to specific interference. Competition or competitive behaviors can also affect the plant at various organizational levels resulting in morphological responses (plant growth), biochemical responses (plant defense) and resource allocation (Novoplansky, 2009; Yamawo, 2015). A better understanding of these phenotypic responses is then critical to better manage vegetation composition and dynamics.

This trade-off between plant growth and defense (also called “the dilemma of plants”) has been often discussed but is not currently well understood (Ballhorn et al., 2014). The growth-defense dilemma is a central paradigm in plant biology, but it is generally analyzed in the context of plant herbivory with numerous hypotheses associated with resource allocation including the “optimal defense,” “carbon-nutrient balance,” and “growth differentiation hypotheses” (Herm and Mattson, 1992; Stamp, 2003; Agrawal, 2007). However, this trade-off is less well-described in the context of complex plant interactions (Lankau and Kliebenstein, 2009; Pierik et al., 2013). In this context, the compensatory continuum hypothesis predicts that plants growing under reduced competition will allocate more resources to defense than under highly competitive conditions because the development of defenses associated with anti-herbivory is most costly under competitive conditions (Cipollini, 2007, 2010). In contrast, the defense stress benefit hypothesis predicts that additional beneficial functions of defensive traits will emerge under competition, and these include allelopathy associational defenses (Inderjit and Del Moral, 1997; Lankau and Strauss, 2007). To date, several studies have documented the increase of secondary compounds or changes in chemical profile in response to the presence of neighboring plant species (i.e., competition, Barton and Bowers, 2006; Jones et al., 2006; Thorpe et al., 2011; Lankau, 2012) or upon exposure to specific allelochemicals or signaling molecules (i.e., allelopathy, Metlen et al., 2009; Xu et al., 2010; Scognamiglio et al., 2014). However, to our knowledge no study has evaluated response to both interference mechanisms, competition and allelopathy, to determine their relative importance with respect to the induction of secondary metabolites in receiver plants, particularly in a forest ecosystem. Metabolic profiling or metabolomic approaches offer particularly strong tools to gain insight into impacts of biotic stress on plant regulation and metabolism, as they relate to plant defense (Scognamiglio et al., 2015; Weston et al., 2015). Such an ecometabolomic approach could provide meaningful information about the physiological mechanisms plants use to respond to numerous stressors in terrestrial communities. In addition, this approach will facilitate the analysis of species-specific responses to plant-plant interferences encountered; in this case resource competition (C), allelopathy (A), or the combination of both processes (AC) (Hartley et al., 2012; Scognamiglio et al., 2015).

The Mediterranean tree *Pinus halepensis* L. has been the subject of recent studies because this species typically colonizes post agricultural/fire open lands and forms dense monospecific mature stands. Mature *P. halepensis* woodlands show limited regeneration of pine seedlings in the absence of any disturbances (Prévosto et al., 2015) counterbalanced by a greater regeneration of *Quercus pubescens* Willd., a late successional species (Lookingbill and Zavala, 2000). *Pinus* is known to produce large quantities of secondary metabolites including phenolics and mono- and sesquiterpenoids which can induce allelopathic responses and alter plant community composition (Fernandez et al., 2006, 2013) and ecosystem functioning (Chomel et al., 2014; Santonja et al., 2015). Recent studies showed that *P. halepensis* aqueous needle extracts strongly inhibited germination and growth of *P. halepensis* seedlings (Fernandez et al., 2008; Monnier et al., 2011). Secondary products may affect *P. halepensis* competitive abilities, and could also contribute to the regenerative success of *Q. pubescens* in the *P. halepensis* understory (Fernandez et al., 2008).

However, field assessment of allelopathic interference remains challenging because of the methodological difficulties associated with investigations concerning allelopathy. It is also particularly difficult to separate allelopathic interference from competition in studies with perennial or aquatic plants (Olofsdotter et al., 1999). Therefore, we designed a greenhouse-controlled environment experiment to further examine both allelopathy (A) and competition (C) in order to better explain the regenerative success of pine seedlings over oak seedlings in pine forests. Our objective was to evaluate the impact of allelopathy (A) (i.e., exposure to aqueous extracts of *P. halepensis*) and competition (C) (i.e., presence of neighbors) on competitive (i.e., growth) and defensive (i.e., secondary metabolite production) traits of *P. halepensis* and *Q. pubescens*. More specifically we asked the following questions: (i) Do allelopathy and competition affect the growth and defensive abilities of target plant species in a similar manner? (ii) Do allelopathy and/or competition impact specific chemical defenses? (iii) Are response interference mechanisms observed species specific? and finally (iv) is there a trade-off between growth and defense in response to allelopathy and/or competition for resources?

## Materials and methods

### Experimental site and design

This study was conducted over a 2-year period (from May 2006 to July 2008) in an experimental plant nursery located near Aix-en-Provence, southern France (43°30′N, 5°24′E). The local climate was meso-Mediterranean, experiencing cool to cold winters and marked summer drought. Mean annual rainfall was 620 mm (Aix-en-Provence Weather station, 1961–1996) and mean monthly temperatures ranged between 5.8°C in January to 22.1°C in July.

*P. halepensis* (hereafter *Pinus*) and *Q. pubescens* (hereafter *Quercus*) seeds were harvested in a Mediterranean forest near the experimental site. In May 2006, the experiment was established with 1-year-old nursery-grown *Pinus* and *Quercus* seedlings of uniform size arising from germinated seedlings and transplanted in 10 l plastic pots. We used a common well-drained soil mixture consisting of 25% calcareous sand, 25% siliceous sand, and 50% mineral soil from “Granulat Provence®”. This soil was used as the growth medium in order to alleviate any chemical inhibition associated with the use of an organic substrate. The seedlings were grown outdoors and regularly drip irrigated to prevent water stress over the course of the experiment. Fertilizer was applied once per week with irrigation (375 mg N, 42.5 mg P and 103.7 mg K) at levels found to be non-limiting for plant growth. All pots were placed under a shade cloth so as to reproduce light conditions similar to those encountered under a dense pine forest canopy, with approximately 80% light interception (Broncano et al., 1998; Maestre and Cortina, 2004; Monnier et al., 2011).

**Figure 1:**
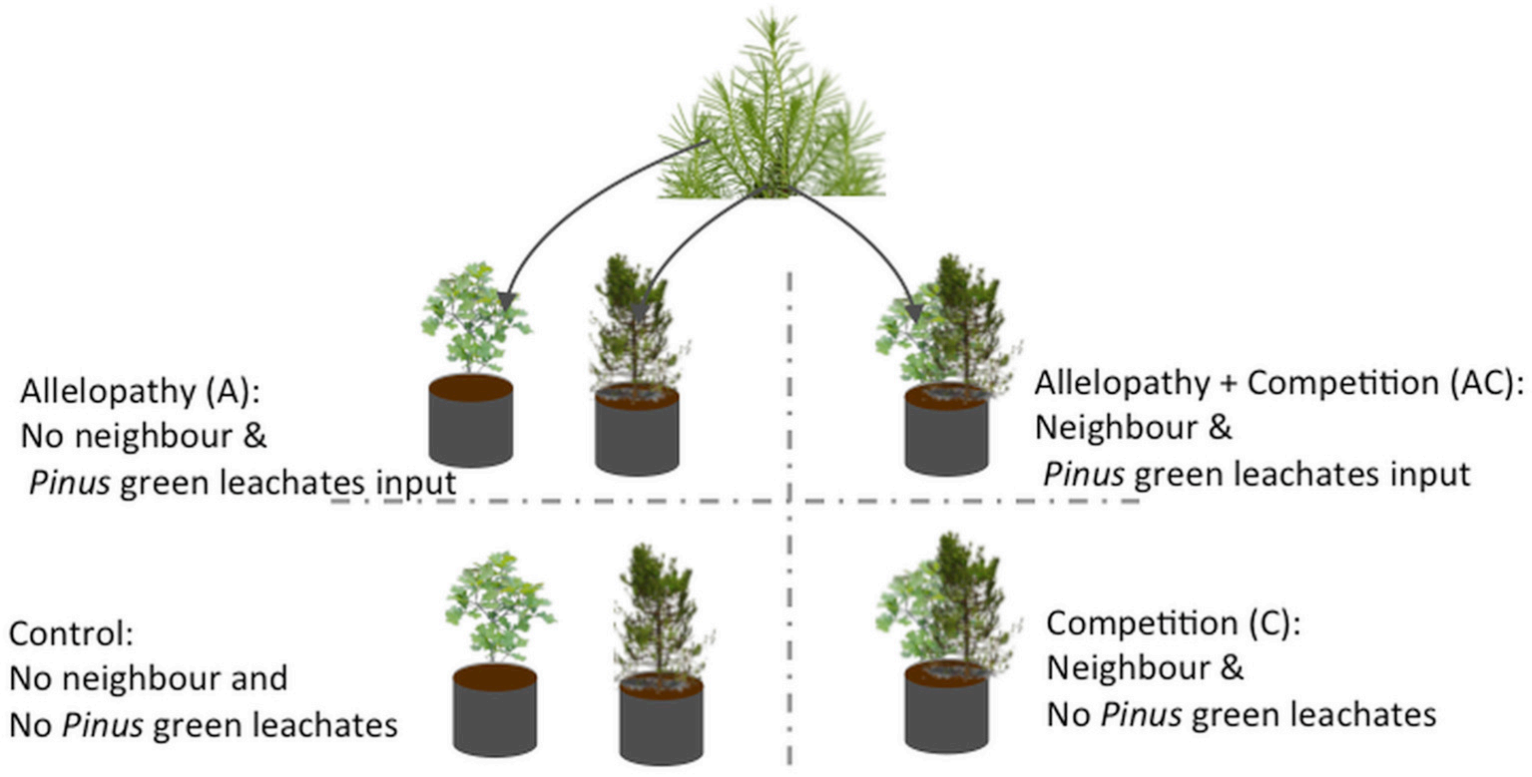
**Diagram of factorial design utilized for experimentation**. Three interference treatments and one control were tested on *Pinus* and *Quercus* saplings: allelopathic treatment was applied by application of *Pinus* needle aqueous extract; competition treatment through neighbor presence; allelopathy + competition treatment through neighbor presence plus application of *Pinus* needle aqueous extract; and control treatment.

A replicated factorial experiment in which saplings of *Pinus* and *Quercus* were exposed to three interference treatments in comparison to one control treatment was conducted with 20 replicates of each species per treatment (*n* = 160 pots). Interference treatments included: (i) allelopathy (A) mimicked by monthly watering of saplings with 0.5 l of aqueous pine needle extracts from mature *Pinus* trees; (ii) competition (C) conditions simulated by co-locating one sapling with a neighboring sapling of the other species in the same pot; (iii) a combination of the two previous treatments (i.e., allelopathy + competition AC) where neighboring saplings were co-located in the same pot (one sapling of each species) and irrigated with 0.5 l of aqueous needle extract; (iv) and a control treatment, where saplings were grown alone and irrigated with 0.5 l water (Figure 1).

To simulate allelopathic interference, the use of aqueous extracts is particularly relevant for assessment of the joint action of mixtures of metabolites rather than a single metabolite of interest (Inderjit and Nilsen, 2003; Fernandez et al., 2008). Aqueous extracts of *Pinus* needles were used to simulate leaf leachates from a forest canopy that could potentially be important in chemically-mediated forest interactions (Mallik, 2008). To simulate competition (C, one *Quercus* + one *Pinus* in the same pot), the physical proximity of the root systems of both species was critical and therefore both species were co-located in the same potting container. Thus, co-location of both species could also generate chemical interference due to the release of allelochemicals from neighboring root exudates. A previous study evaluating *Pinus halepensis* growth over time revealed limited allelopathic potential associated with root extracts obtained from young seedlings (Fernandez et al., 2006). Therefore, the effect of root exudates released by small saplings in this experiment is likely to be negligible compared to interference associated with resource competition.

To prepare aqueous extracts of *Pinus* needles for later application to pots, 25 kg of needles were collected from a pine forest (*circa* 20 years old) near Aix-en-Provence throughout the growing season, generally on a monthly basis. Fresh needles were consistently macerated in 250 l of water for 48 h, in dark conditions (Yu et al., 2003) in order to obtain leachate concentration of 10% fresh weight, corresponding to 5% dry weight (Fernandez et al., 2006). Irrigation was performed just after maceration. Preparation of aqueous extracts for irrigation of pots was performed monthly.

At experimental termination, soil carbon and nitrogen content were analyzed in order to be certain that target pots were not enriched by N-containing compounds present in the aqueous extract (*t*-test, *P* > 0.05).

### Plant phenotypic responses

In July 2006, at experimental initiation, *Pinus* and *Quercus* sapling traits (height and basal diameter) were measured prior to treatment application, and no significant differences were observed for either of these two variables. Height and basal diameter were assessed in *Pinus* and *Quercus* saplings commencing in winter 2007 until summer 2008, at four specific dates (February 2007, May 2007, March 2008, and July 2008). *Pinus* height was determined as the length from the stem collar to highest apex. In *Quercus*, as most individuals were multi-stemmed with no clear leader shoot, the cumulative length of all stems was measured. At experimental termination, each sapling was excavated and transported to the laboratory where separation into roots, stems and leaves was performed. As it was not possible to separate root systems of the two species in the C treatment due to extreme intertwining of both root systems, roots were not weighed. After processing, all samples were dried at 60°C for approximately 72 h, after which the dry mass of each sample was recorded.

Once harvested, plant phenolics were estimated both qualitatively and quantitatively at leaf level at experimental termination as total phenolic concentration and composition in samples of *Pinus* and *Quercus* foliage. Terpenoid composition was also estimated for *Pinus* samples. At the leaf level, total phenolic concentrations were determined based on the Folin method described by Singleton and Rossi (1965). Individuals of both species (3 < *n* < 9) were sampled on the same date in July 2008 by harvesting similarly aged leaves located in similar positions on the crown. One-half g (dry weight) of leaves or needles per sample was extracted at room temperature for 1.5 h by gentle shaking in a 70% (v/v) aqueous methanol solution (20 mL) acidified with a few drops of 1N HCl and filtered. Quantification of total phenolics was performed by colorimetric reaction using the Folin-Ciocalteu reagent. After 1 h, the reaction was completed and measured at 720 nm on a spectrophotometer (Biomate3, Thermofisher). Quantitative results were expressed in mg of gallic acid equivalent g^−1^ dry weight.

Further, a targeted metabolomic approach was used to assess plant metabolites present in sample extracts in which primary (mostly aliphatic acids) and secondary (i.e., terpenoids and phenolics) leaf metabolites were investigated as per Fernandez et al. (2009). Both polar (fatty acids, fatty diacids, simple phenols, acetophenones, phenolic acids, and cinnamic acids), and less polar metabolites (monoterpenes and sesquiterpenes) were quantified using GC-MS instrumentation (Hewlett-Packard GC6890 coupled to a HP5973N Mass Selective Detector equipped with a HP-5MS capillary column (30 m × 0.25 mm × 0.25 μm—J&W Scientific)). A specific SIM (Selected Ion Monitoring) method was developed to analyze polar metabolites by determination of molecular features including fragment ions and retention time of injected authentic reference standards (Sigma-Aldrich®). A SCAN method was developed for less polar compounds analyzed. Positive identification was performed by comparison of MS spectra to those of authentic reference standards (Sigma-Aldrich®). Database searches in the NIST 2008 mass spectral library were conducted to tentatively identify major constituents. Retention indexes of compounds were determined relative to Wisconsin Diesel Range Hydrocarbon injection (Interchim, Montluçon, France) and tentatively confirmed by comparison with those reported in the literature (Adams, 2007). Concentrations were expressed in mg g-^1^ of dry weight. Phenolic and terpenoid allocation refers to the ratio between total phenolic content (Folin method) or total terpenoid content (sum of all terpenoids analyzed by GC/MS) and carbon content (CHN analyser).

### Data analysis and overall phenotypic responses

After checking ANOVA assumptions, repeated measures two-way ANOVA, followed by Tukey tests for post hoc pairwise comparisons, were performed to study temporal effects of each treatment on whole plant response variables (height, diameter) at the within species level. One-way ANOVA, followed by Tukey tests for post hoc pairwise comparisons, were performed to study the effects of each treatment on aerial biomass at the end of the experiment. Belowground biomass and belowground allocation were assessed between Control and Allelopathy treatments using two-tailed student *t*-tests.

One-tailed student *t*-tests were performed to test the hypothesis of higher concentrations/allocations of phenolics and terpenoids in interference treatments in comparison to the control. In the case of unequal variance, unpaired one-tailed *t*-tests with Welch's correction were conducted. Variation in chemical composition by treatment was analyzed by using Principal Component Analysis (PCA) centered and scaled to unit variance. Differences in the concentration of each compound between interference treatments and control were tested with the Mann-Whitney tests. Similarity percentages (SIMPER analysis) were performed in order to identify the molecular features for which the variations contribute most to the dissimilarity between control and interference treatment responses.

Phenotypic plasticity has gained increasing attention with the need to predict species responses to global climate change (Richter et al., 2012). Several metrics have been proposed to assess this environmental source of variability (Valladares et al., 2006). In the present study, we employed the phenotypic plasticity index (PI), a metric recommended to explore functionally related traits for variables with different units and with contrasting ranges. PI is based on maximum and minimum trait means across environmental conditions and was calculated for every trait and species as: (trait mean among treatment (A, C, or AC)—trait mean among control))/max trait mean (treatment or control; Valladares et al., 2006). The index scales from -1 to 1 where an index value close to 0 indicates an absence of response to the treatment. Inversely, an index value close to 1 or -1 indicates a strong response to the treatment. Positive or negative value of PI for a trait indicates respectively positive or negative phenotypic response of this trait to corresponding interference treatment. Further we represented neighbor-defensive behavior with an overall phenotypic response (OPR) by representing side by side PI of seven traits (terpenoid content, terpenoid allocation, phenolic content, phenolic allocation, height, diameter and aerial biomass) for each species in each treatment. Visualizing profiles of OPR for each species enabled the comparison of behavioral strategies among species in response to different treatments. The within-species shifts in behavioral sensitivity when facing two different treatments were then assessed through correlations between OPRs to each treatment. Similarly, the between-species variability of OPR was assessed through Spearman correlations between the OPR of both species to the same treatment. In order to further examine the trade-off between growth and defense, PCA was performed with all traits measured (height, diameter, aerial biomass, terpenoid content, terpenoid allocation, phenolic content, phenolic allocation).

Univariate analysis (*t*-tests, ANOVA, Tukey tests, Mann-Whitney tests) and regression analysis were performed using R Studio software (version 0.99.483, 2009–2015, R Studio, Inc.); Multivariate analysis (PCA and SIMPER analysis) were performed using PRIMER-E software (Plymouth Routines in Multivariate Ecological Research, version 6.1); OPR representation were realized using GraphPad software (GraphPad Prism version 5.00 for Windows).

## Results

### Growth response to competition and allelopathy

**Figure 2:**
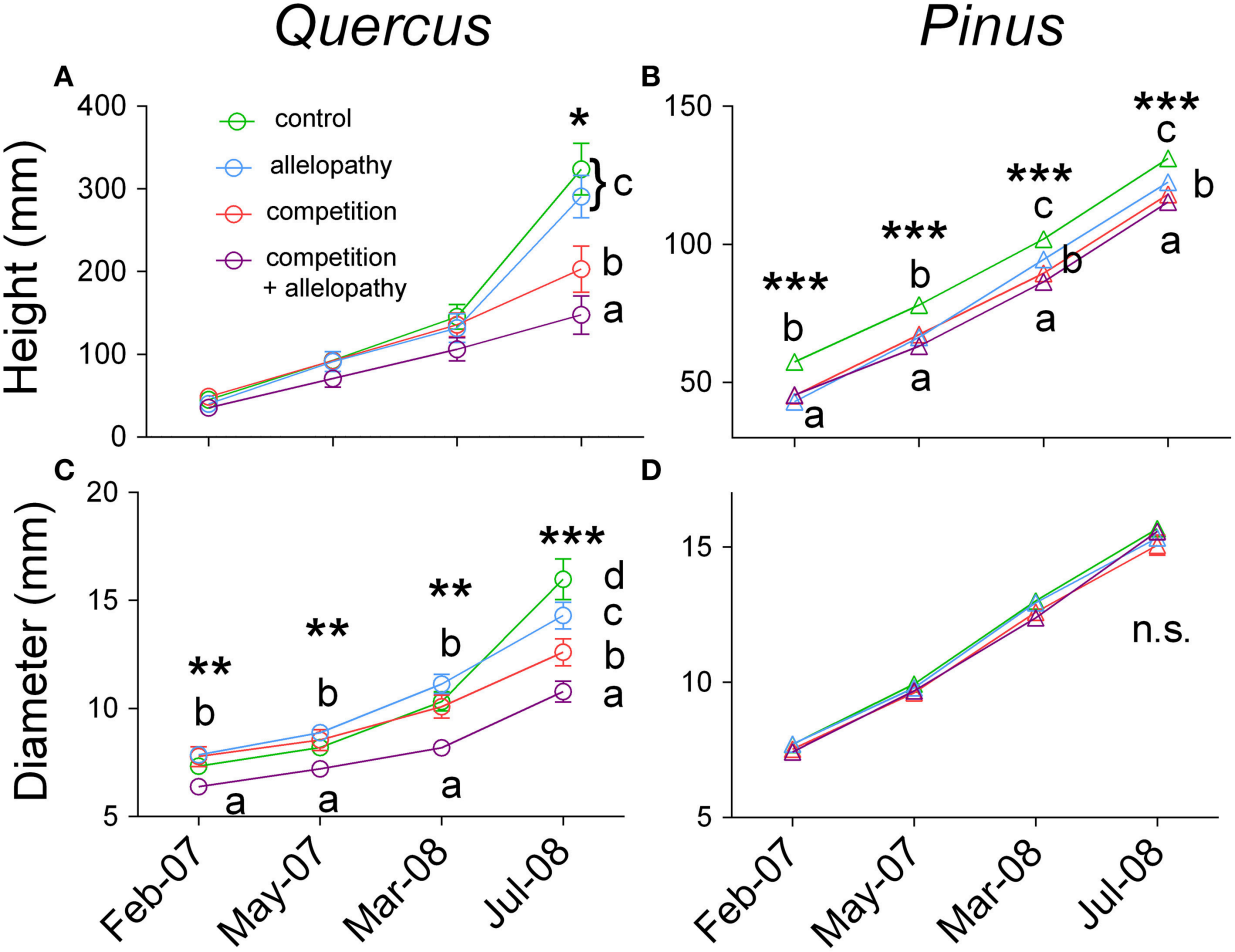
**Temporal effects of interference treatment on growth of both species**. Height **(A,B)** and diameter growth **(C,D)** response in Quercus **(A–C)** and Pinus **(B–D)** across four dates of measurements (February and May 2007, March and July 2008) in Control (green lines and symbols), Allelopathy (blue lines and symbols), Competition (red lines and symbols), and Allelopathy + Competition (purple lines and symbols). Symbols represent means ± SD of 20 replicates. Different letters indicate a significant difference between treatments at *P* < 0.05 (^*^), *P* < 0.01 (^**^), and *P* < 0.001 (^***^).

**Figure 3:**
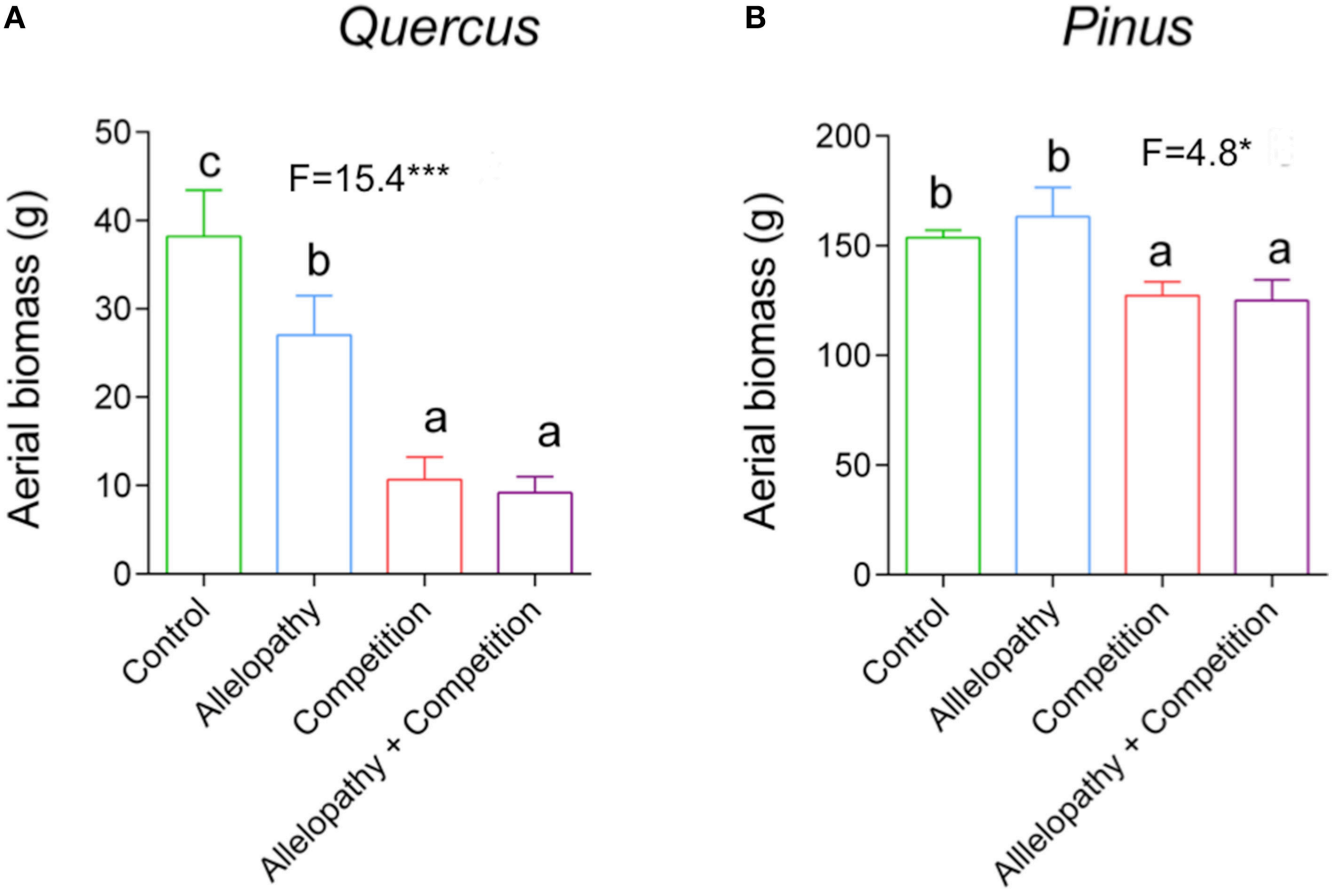
**Effects of interference treatment on aerial biomass of ***Pinus*** and ***Quercus*****. Height growth response in *Quercus* **(A)** and *Pinus* **(B)** in Control (green bars), Allelopathy (blue bars), Competition (red bars), and Allelopathy + Competition (purple bars) treatments. Bars are means ± SD (5 ≤ N ≤ 12). Different letters indicate a significant difference between treatments.

**Figure 4:**
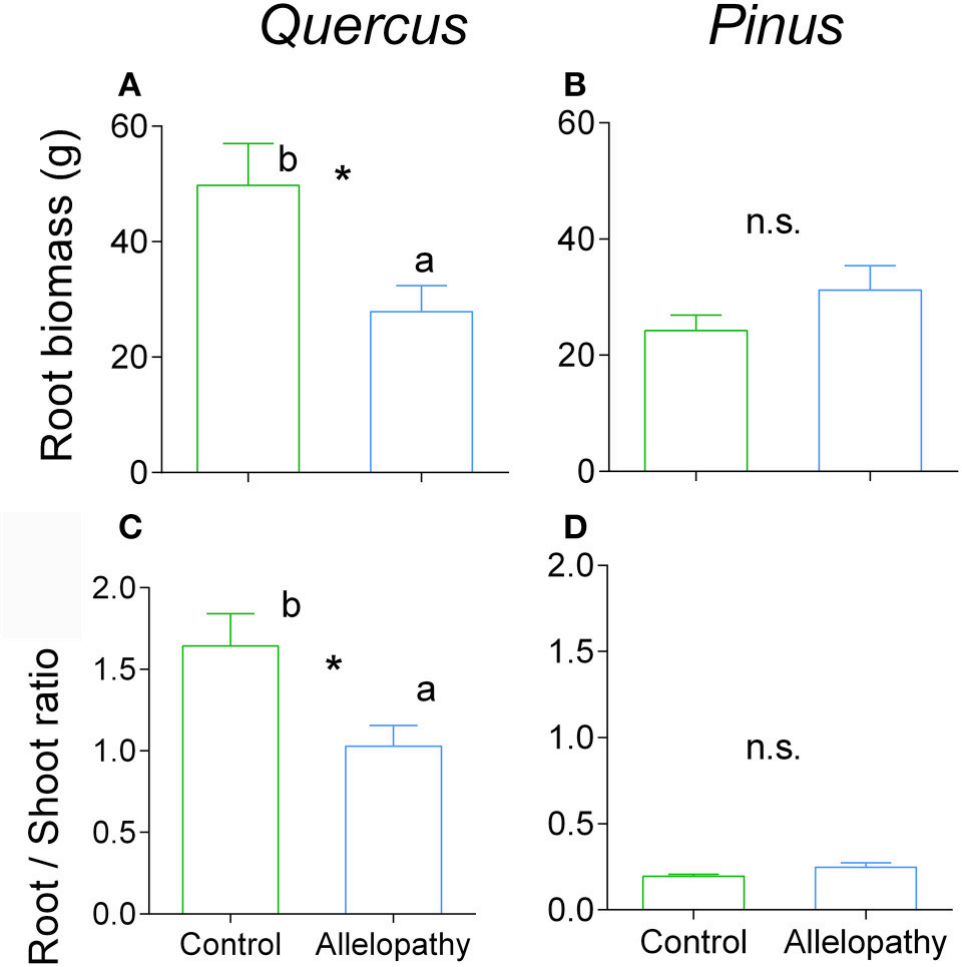
**Effects of allelopathic treatment in belowground biomass and allocation**. Belowground biomass in Quercus **(A)** and Pinus **(B)** and belowground allocation in Quercus **(C)** and Pinus **(D)** in Control (green bars) and Allelopathy (blue bars) treatments. Bars are means ± SD (5 ≤ *n* ≤ 8). Different letters indicate a significant difference between control and allelopathy treatments at *P* < 0.05 (^*^).

Growth of *Pinus* and *Quercus* saplings (i.e., diameter and height) was significantly affected by all three interference treatments with increasing significance of these effects over time (two-way ANOVA; Table 1). All treatments affected *Pinus* height similarly during the first year: 78 mm height was observed in the control and 66, 67, and 63 mm was observed in A, C, and AC treatments respectively, corresponding to a decrease in growth of approximately 16% in the interference treatments. At experimental termination, height was significantly decreased for C and AC treatments in comparison to A treatment (Figure 2B). No treatment effect was noted for *Pinus* diameter readings (Table 1; Figure 2D). Aerial biomass of *Pinus* saplings was inhibited by C (-17%) and AC (-19%) treatments (Figure 3B). A treatment did not affect aerial (Figure 3B) and root (Figure 4B) biomass of *Pinus* saplings, but altered biomass allocation, resulting in a slight increase in belowground allocation of resources (i.e., increase of root/shoot ratio; Figure 4D).

**Table 1:** **Temporal effects of interference treatments on growth traits of ***Pinus*** and ***Quercus*****.

**Factors**	**DF**	**Quercus**				**Pinus**			
		**Diameter**		**Height**		**Diameter**		**Height**	
		**F**	***P*-value**	**F**	***P*-value**	**F**	***P*-value**	**F**	***P*-value**
Time	3	252.4	<**0.001**	182.7	<**0.001**	1618.0	<**0.001**	2558.0	<**0.001**
IT	3	7.1	<**0.001**	4.3	<**0.01**	0.9	0.418	12.0	<**0.001**
Time^*^IT	9	7.6	<**0.001**	8.5	<**0.001**	0.9	0.497	3.5	<**0.001**

*Summary of the repeated measures ANOVA for growth in diameter and height in response to interference treatments (IT) in Quercus and Pinus. Significant P-values are typed in bold (n = 20)*.

All interference treatments reduced *Quercus* height at experimental termination. Height ranged from 324 mm for the control to 291 mm for A (−10%), 203 mm for C (−37%), and 148 mm for AC treatments (−54%) (Figure 2A). The effect of interference treatments on *Quercus* diameter was similar and followed the same trends. AC treatment resulted in reduced *Quercus* diameter throughout the experiment whereas A and C treatments decreased diameter significantly only at the last sampling date. At this time, the diameter was reduced from 16 mm in the control to 11 mm (−33%) for AC, 13 mm for C (−21%), and 14 mm for A (-11%) (Figure 2C). Similarly to other growth parameters assessed, aerial biomass of *Quercus* saplings was reduced by A (−29%), C (−71%), and AC (−76%) treatments (Figure 3A). Allelopathic interference in the A treatment resulted in a 50% decrease in *Quercus* root biomass with 28 g root biomass observed in A treatment in contrast to 50 g in the control (Figure 4A). Biomass allocation was also altered by A treatment leading to a strong decrease in belowground resource allocation (i.e., decrease in root/shoot ratio; Figure 4C).

### Biochemical responses to neighbor presence and allelochemical exposure

*Pinus* responded to A, C, and AC treatments by increasing total terpenoid content (except for allelopathy) and terpenoid allocation (Table 2). For *Pinus*, a species known to produce high concentrations of terpenoids and phenolics, 40 terpenoids, and 19 polar compounds (see Supplementary Tables S1, S2 for more details) were identified. The most abundant terpenoids included α-pinene (monoterpene; up to 225 μg.g^−1^DW) and β -caryophyllene (sesquiterpene; up to 448 μg.g-^1^DW) and gallic acid was most abundant with respect to phenolic acids (up to 1468μg.g^−1^DW).

**Table 2:** **Species-specific (***Quercus*** and ***Pinus*** separately) and overall (mean of both species) effects of treatments on induced-secondary metabolism**.

	**Control**	**Allelopathy**	**Competition**	**Competition + Allelopathy**
**QUERCUS**				
Phenolic content	16.51 ± 5.94	22.88 ± 10.30	23.17 ± 3.91	25.69 ± 3.52
Phenolic allocation	0.34 ± 0.10	0.55 ± 0.16	0.51 ± 0.08	**0.55** ± 0.07^*^
**PINUS**				
Phenolic content	18.51 ± 2.86	20.04 ± 3.24	22.49 ± 1.96	25.71 ± 4.68
Phenolic allocation	0.31 ± 0.04	0.37 ± 0.05	0.45 ± 0.04	0.53 ± 0.10
Terpenoid content	1.48 ± 0.31	1.70 ± 0.33	**2.76** ± 0.64^*^	**2.80** ± 0.59^*^
Terpenoid allocation	0.018 ± 0.003	**0.023** ± 0.005^*^	**0.043** ± 0.001^*^	**0.042** ± 0.001^*^
**BOTH SPECIES**				
Phenolic content	17.65 ± 2.71	**21.46** ± 4.87^*^	**22.78** ± 1.82^*^	**25.69** ± 2.59^*^
Phenolic allocation	0.33 ± 0.05	**0.46** ± 0.08^*^	**0.48** ± 0.04^*^	**0.55** ± 0.05^**^

Phenolic (Folin method) and terpenoid (sum of all terpenes measured by GC-MC method) contents are expressed as mg. g^−1^ dry weight (mean ± SE) and allocation of phenolics and terpenoids as ratio of carbon content (mean ± SE). One-tailed student t-tests were performed to test the hypothesis that means of content and allocation are significantly higher with interference treatments than control treatment. Asterisks indicate a significant higher value compared to the control treatment at P < 0.05 (^*^) and P < 0.01 (^**^). Significant P-values are typed in bold.

**Figure 5:**
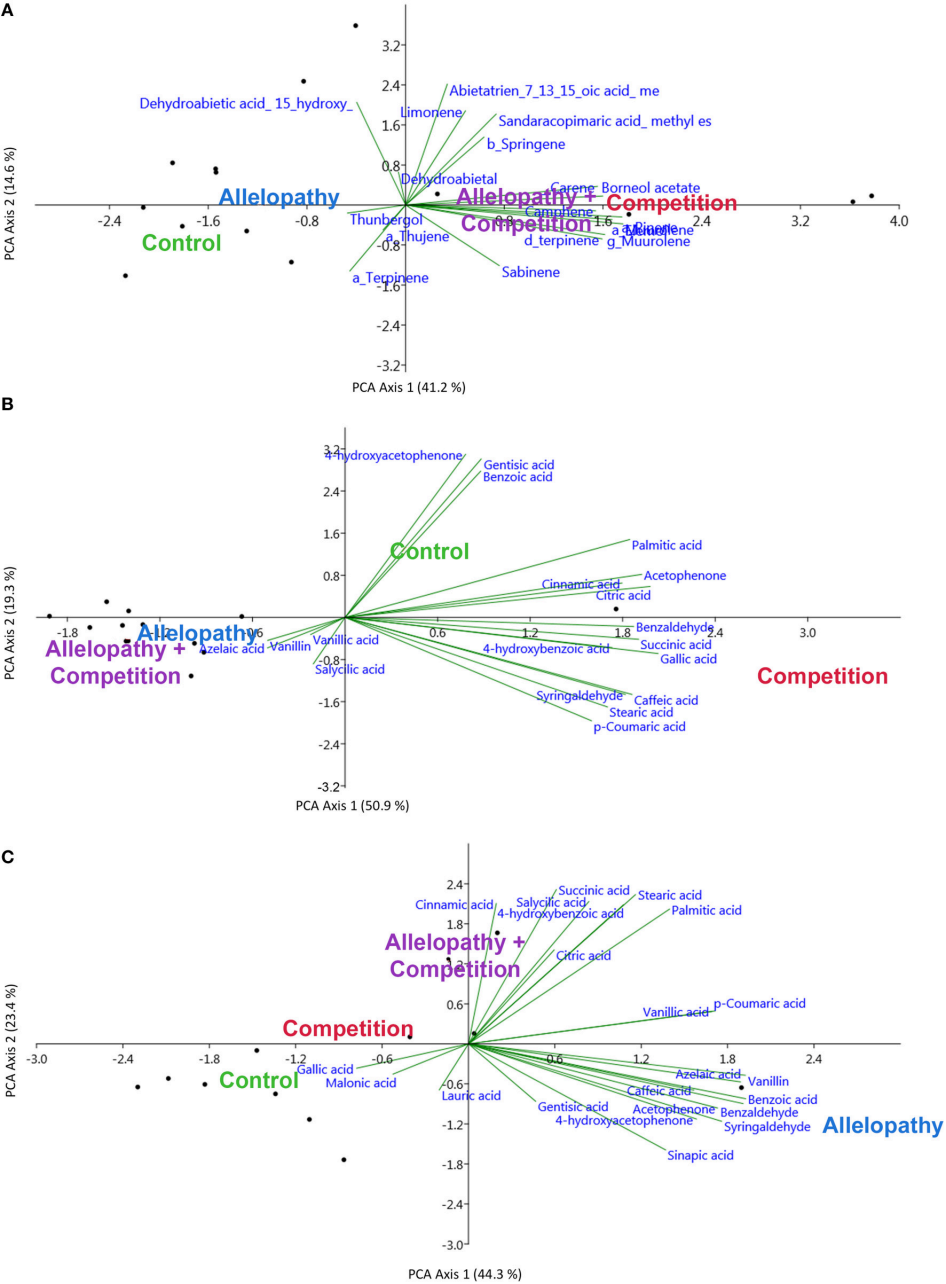
**Principal component analyses performed for metabolites most closely associated with responses to A, C, and AC (SIMPER analysis), for terpenoids for ***Pinus*** (A) and for polar metabolites in ***Pinus*** (B) and ***Quercus*** (C)**.

PCA revealed considerable variation in terpenoid profiles in *Pinus*, particularly in regards to C and AC treatments. Figure 5A; Camphene was clearly induced by C treatment, and this metabolite was not observed in any of the control samples (Mann-Whitney tests, 0.05 < *P* < 0.10; PCA). Its presence accounted for much of the variation or dissimilarity between the control and C treatment (SIMPER analysis). δ3-carene, α-pinene, terpinene, ß-caryophyllene, elemol and and δ-germacrene concentrations increased respectively by a factor of 5–30 (Mann-Whitney tests, 0.05 < *P* < 0.10) in A and AC treatments but the first 3 compounds alone accounted for the much of the dissimilarity with control (SIMPER, Supplementary Table S3).

After analysis of *Pinus* polar compounds, 4-hydroxyacetophenone was detected in the control and was not observed in other treatments (Mann-Whitney tests, *P* < 0.05), and this contributed largely to dissimilarity between control and all other treatments (SIMPER analysis, Supplementary Table S3). Gallic acid, citric acid, and acetophenone were mainly present in treatment C (PCA, Figure 5B), but in A were decreased in comparison to the control by factors of 5, 10, and 100 respectively (Mann-Whitney tests, *P* < 0.05). Vanillin and gentisic acid decreased while salicylic acid increased in treatment A, and these three compounds explained the majority of the difference between the control and A. Vanillic acid was present in high concentrations in AC treatment, which were increased over the control by a factor of 3, whereas caffeic acid increased by a factor of 10 in C extracts (Supplementary Table S2) and its presence accounted for much of the variation between the control and C (Supplementary Table S3).

*Quercus'* responses to interference treatments revealed a trend toward increased phenolic content and allocation with interference in comparison to the control. Specifically, the AC treatment showed enhanced phenolic production in comparison to the control (Table 2). In the polar extracts, over 22 compounds were identified and citric and gallic acids were the two most abundant metabolites. PCA also revealed differentiation in *Quercus* polar metabolic profiles (Figure 5C). In this case 4-hydroxyacetophenone and vanillin were found in higher abundance in A in comparison to the control whereas salicylic and 4-hydroxybenzoic acid were found in greater abundance in treatment C and AC (Supplementary Table S2, Mann-Whitney test, *P* < 0.10; Supplementary Table S3, SIMPER analysis).

### Species-specific patterns of overall phenotypic response (OPR)

**Figure 6:**
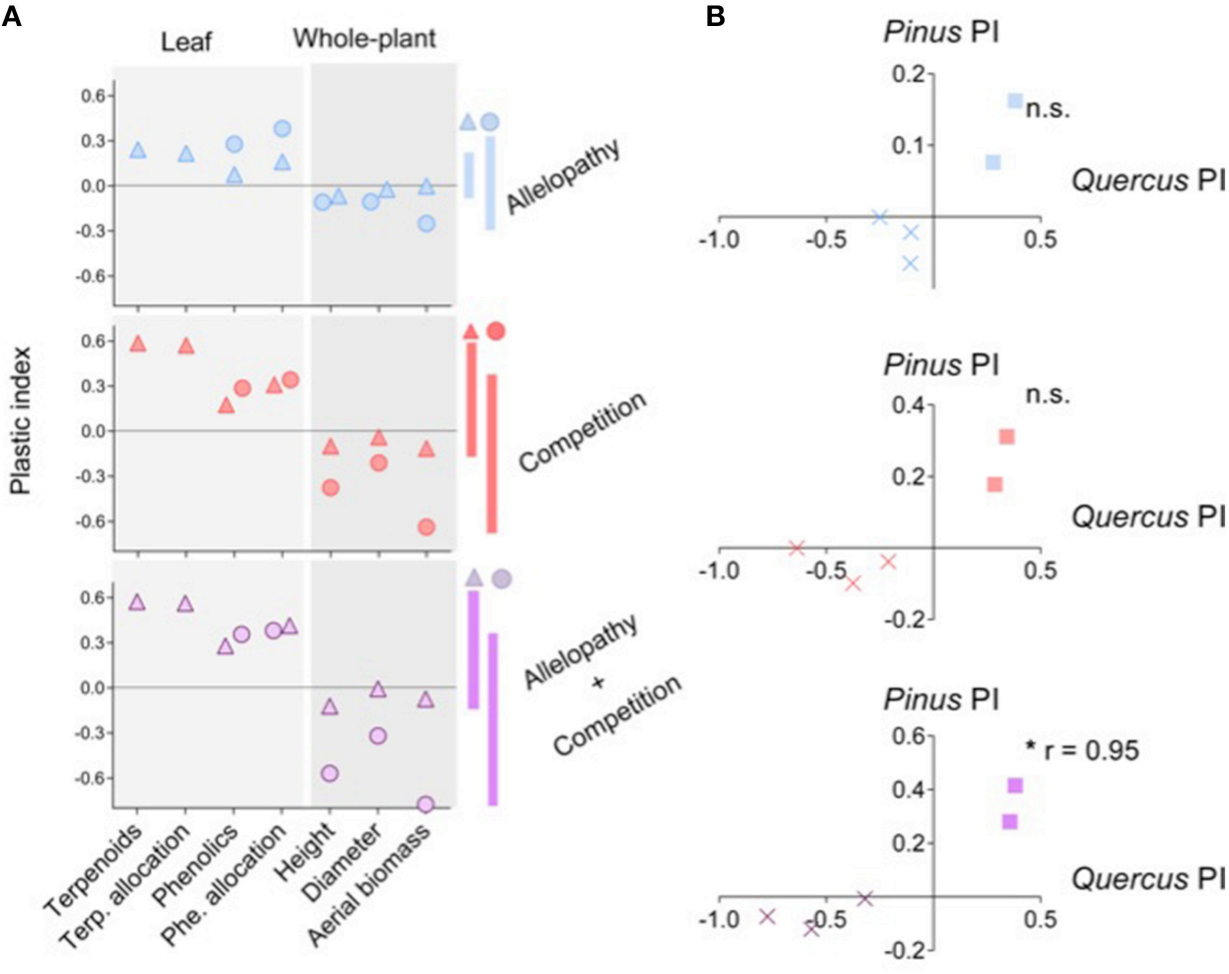
**Overall Phenotypic Responses (OPR) of ***Pinus*** and ***Quercus*** to the three interference treatments**. Global representation **(A)** of plastic index (PI) of 7 selected traits in response to Allelopathy (blue symbols), Competition (red symbols) and Allelopathy + Competition (purple symbols) treatments for *Pinus* (triangles) and *Quercus* (circle). Positive or negative value of PI for a trait indicates positive or negative phenotypic response, respectively, for this trait to corresponding interference treatment. Vertical bars indicate the magnitude of the OPR in both species to each interference treatment. Pearson correlation **(B)** between the OPR (cross for growth traits and square for biochemical traits) of both species to Allelopathy (blue symbols), Competition (red symbols) and Allelopathy + Competition (purple symbols) treatments. Asterisks indicate a significant value at *P* < 0.05 (^*^).

In general, both species presented a similar OPR when exposed to interference treatments, with enhanced production of secondary metabolites and reduced overall growth as assessed by measurement of various growth traits (Figure 6A), showing a clear trade-off between growth and defense abilities (PCA analysis, Supplementary Figure S1). However, *Pinus* had less overall growth reduction than *Quercus* but exhibited a stronger biochemical or plant defense response when subjected to interference treatments (Figure 6A). The OPR to various interference treatments was more highly correlated for *Quercus* saplings (0.94 < *r* < 0.99) than *Pinus* saplings (0.76 < *r* < 0.91), which could be interpreted as a less specific response for *Quercus* saplings than for *Pinus*. OPR patterns in A and C differed among the two species, but were positively correlated in response to AC treatment (*r* = 0.95, *P* = 0.01; Figure 6B).

## Discussion

### Growth is more affected by competition than allelopathy

This study has shown that both allelopathy and competition are plant interference mechanisms that can impact the growth of the perennial forest species under evaluation in a variable manner. Specifically, a hierarchical response to the *Quercus* competition significantly affected height, diameter and aerial biomass more than did allelopathic interference and a cumulative effect was observed when the two mechanisms were combined, suggesting that allelopathy renders *Quercus* more susceptible to competition, as previously reported by Viard-Crétat et al. (2012). *Pinus* was less affected by allelopathic interference than was *Quercus* as only the *Pinus*' height decreased in response to treatment of plant extracts. Autotoxicity was not specifically observed with regards to the growth parameters of *Pinus* saplings (trees with diameter > 2.5 cm) in contrast to *Pinus* seedlings (early stage of life just after germination) which exhibited strong potential autotoxicity, both in germination and early growth (Fernandez et al., 2008). These findings suggest the importance of ontogeny on the allelopathy process as different life stages exhibited differential sensitivity to allelopathic interference.

### Defense response to plant interference is highly species-specific and is more affected by competition than allelopathy

**Figure 7:**
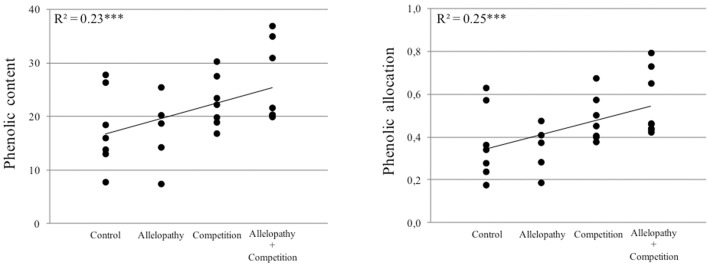
**Relationship between phenolic content or phenol allocation and the gradient of interference for both ***Pinus*** and ***Quercus*** species (Control, Allelopathy, Competition, Allelopathy + Competition)**.

Plant interference treatments induced changes in production and allocation of chemical defenses, assessed by measurement of secondary metabolites, in both species evaluated. For *Pinus* and *Quercus*, total phenolic content and allocation to plant chemical defense increased according to the following gradient “Control < Allelopathy < Competition < Allelopathy + Competition” (Figure 7). For *Pinus*, competition resulted in the induction of higher terpenoid content than did allelopathy and no cumulative effect with combined interference mechanisms was observed. Ormeño et al. (2007a) reported an increase in terpenoid content with increasing competition for resources in *Pinus*. It should be noted that this increase was species dependent. Our findings demonstrated that plants may initiate a defensive response through chemical detection of neighbors in the absence of physical cues (allelopathy treatment with no direct contact with competitor), similar to those well-described findings for animal-defensive behavior (Callaway, 2002) or against abiotic stress (Ormeño et al., 2007b). Additionally, the magnitude of response to chemical signaling is evidently dependent or associated with a cumulative effect of various interference mechanisms, i.e., differential induction of chemicals in plants exposed to allelochemicals, competition for resources and combined interference (accumulation of chemical and physical cues). The differential response pattern observed could potentially be further explained by the diversity and amount of competing signals (root exudates, volatile compounds, physical contact) perceived in the case of the presence of an interfering neighbor, in addition to the complex mixture of compounds or chemical signals released upon plant exposure by application of leachates. These results also suggest that plants can potentially modulate their chemical responses or biosynthetic pathway regulation in response to different biotic stressors or interference mechanisms (Broz et al., 2010).

The production of diverse classes of metabolites (including terpenoids, phenolics, and aliphatic acids) may represent an ecological advantage by favoring induction of the metabolite class most effective against temporal changes in external threats (Goodger et al., 2013). In plant-herbivore interactions, mixtures of secondary metabolites are described as advantageous if various components target several enemies (Gershenzon and Dudareva, 2007; Gershenzon et al., 2012). Our results suggest a similar process occurs in plant-plant interference, with the specific induction of selected terpenoids or phenolics in response to variable stressors or signals.

For phenolics the metabolite 4-hydroxyacetophenone was upregulated specifically in response to allelopathic interference treatment for *Quercus* but this was not observed in *Pinus*. This phenolic metabolite is reported to be phytotoxic and also exhibits anti-herbivory properties (Gallet, 1994; Céspedes et al., 2002; Delvas et al., 2011; Ruan et al., 2011). Previous studies have also described induction of this compound (and of its glycoside picein) in response to biotic stress conditions, but without the influence of a neighbor, which is in agreement with the absence of induction of this metabolite observed in competition treatments (Osswald and Benz, 1989; Vrchotová et al., 2004). Vanillin, an abundant phenolic aldehyde, was induced in response to all interference treatments for *Quercus*, with greatest response observed in the allelopathic interference treatment. This compound was also previously identified as a phytotoxin and was shown to possess antifungal properties (Reigosa et al., 1999). Interestingly, concentrations of salicylic acid increased in the competitive interference treatments for *Quercus*. This metabolite is reported to act as a phenolic hormone by influencing many plant processes including growth, development, senescence, and stress responses (Huot et al., 2014). Accumulation in response to biotic interference could potentially activate defense gene expression (Huot et al., 2014) and thus further induce the defense process and subsequent metabolome adjustment.

For terpenoids, several compounds increased greatly in abundance with interference, specifically competition. While some terpenoids were previously reported to be induced by competition (δ3-carene, α-pinene ß-caryophyllene; Ormeño et al., 2007a), others are known to be associated with allelopathic activity (δ3-carene, α-pinene, Camphene; Kordali et al., 2007). However, terpenoids that are especially abundant in coniferous trees are among the most expensive forms of chemical plant defense, from a metabolic standpoint in terms of energy required for production (Gershenzon, 1994). In *Pinus*, the strong upregulation of terpenoids in response to competitive interference suggests adaptive benefits from upregulation may overcome short-term metabolic disadvantages in costs associated with biosynthesis and eventual storage. Our results highlight a forest plant's ability to modulate their specific metabolic profile and thus impact their subsequent defensive capability, depending on the type of biotic interference.

### Growth defense trade-off and implication for competitiveness

Regardless of the interference combination, increase of phenolic content and decrease of growth trait values observed for *Quercus* could be interpreted as a “growth defense trade-off.” In *Pinus*, this trend was observed only in response to interference associated with competition. In response to allelopathic interference, *Pinus* induced chemical defenses were observed but growth traits were not affected. This trade-off phenomenon has been described extensively in plant-insect or plant-pathogen interactions (for a review see Huot et al., 2014) or in the case of exposure to abiotic stress (Genard-Zielinski et al., 2014) but it has been poorly described for plant-plant interference (allelopathy or competition; Rasher and Hay, 2014). Our results highlight the ability of two forest plant species to respond to competitors by adjusting resource allocation in order to increase their relative competitiveness.

Interestingly, *Quercus* presents a more conservative strategy in acclimation to various competitive environments in contrast to *Pinus*. This is illustrated in this study by a magnitude of response which is: (i) greater for negative plastic responses in growth traits and (ii) lower for induced positive plastic responses in defense traits. The results obtained further support conclusions of previous studies suggesting stronger induced plastic responses of *Pinus* and a more conservative behavior from *Quercus* (Monnier et al., 2013).

The correlation between overall phenotypic responses (Figure 6) of both species revealed differential species responses of *Pinus* and *Quercus* when subjected either to allelopathy or to direct resource-based competition. In the combined AC treatment both species showed a strong correlation to OPR pattern. Results provide further evidence for a common response of both species when subjected to harsher competitive environments (the AC treatment). Although phenotypic plasticity resulting in trait divergence increased the ability of plants to coexist and may be an adaptive response to competition (Burns and Strauss, 2012), present results suggest that global response of competing plants may converge in certain strongly competitive environments.

### Implication for plant mediterranean succession

The findings of this study did not support the assumption that saplings of late-successional species colonizing a pioneer forest understory developed less sensitivity to allelochemicals than the pioneer producer species. One explanation for this pattern may arise from the fact that setting up costly mechanisms of tolerance to chemical interference may be evolutionary disadvantageous in favorable growth conditions or environments (Lankau, 2008). For *Quercus, Pinus* forests with intermediate densities often represent favorable environments, which are considered as “safesites” for *Quercus* establishment, creating partial shading, reducing solar radiation and improving the water availability status (Rodriguez-Calcerrada et al., 2010; Prévosto et al., 2011). Under such conditions, it is likely that allelopathy does not play a strong limiting role for *Quercus* regeneration and that development of physiological tolerance to a neighbor's allelochemicals may be more costly than beneficial. Nevertheless, this sensitivity to *Pinus* metabolites may become disadvantageous for *Quercus* in harsher conditions, such as dense *Pinus* stands where *Quercus* development is limited (Prévosto et al., 2011). In addition, previous studies noted a contradiction between suitable recruitment conditions and appropriate conditions for further *Quercus* sapling growth (Puerta-Pinero et al., 2007; Gomez-Aparicio et al., 2008; Sheffer, 2012). Our results support this observation, describing increasing sensitivity to aqueous extract supply over time, likely consequent to the alteration of root system function. In terms of root growth, α-pinene was observed to inhibit root development (Singh et al., 2006; Pierik et al., 2013). Under these conditions, the adaptive strategy of *Pinus* may be to produce toxic secondary compounds and maintain lower sensitivity to these metabolites than neighboring species during early years of development which evidently provides a competitive advantage. Further studies are required to confirm the relative role played by chemical interference in the dynamics of Mediterranean vegetation communities and forest ecosystems. Studies must clearly address specific environmental conditions in which sensitivity to allelopathy represents an evolutionary and competitive disadvantage in comparison to the presence of co-existing species. The impact of drought on chemical interference mechanisms may also be of particular importance, as Mediterranean ecosystems are predicted to be warmer and drier in the face of a changing climate.

## Conclusions

Results reported for the two Mediterranean tree species, *Pinus halepensis* and *Quercus pubescens*, strongly suggest the existence of differential effects of various biotic interference mechanisms on sapling development, and the need to consider their cumulative or antagonistic effects (allelopathy and competition) in plant community dynamics (Viard-Crétat et al., 2012). The magnitude of the responses observed increased with time and highlighted the cumulative impacts of interference mechanisms, pointing to the necessity to conduct long-term (> 1 year) experiments when studying perennial species, in direct contrast to the short-term experiments usually performed in allelopathy research, which typically do not clearly reveal responses to complex biotic interferences. This study demonstrates that *Pinus* and *Quercus* may be able to adopt different resource allocation patterns in response to a range of biotic interference treatments (allelopathy < competition < allelopathy + competition). Responses observed were species specific but may converge in case of strongly competitive environments (both allelopathy and competition simultaneously).

Further studies are required to determine the mechanisms and adaptive implications of the observed differential sensitivity to mixtures of allelochemicals. Our findings suggest the possibility that the perception of various early competitive interference signals may prime juvenile forest plants to better tolerate strongly competitive environments later.

## Author contributions

CF, YM, BP, and AB designed the research; CF, YM, BP, and AB conducted the research; CF, YM, MS, AS, and VB collected and analyzed the data; CF, YM, MS, CG, LW, BP, VB, and AB wrote the manuscript.

### Conflict of interest statement

The authors declare that the research was conducted in the absence of any commercial or financial relationships that could be construed as a potential conflict of interest.

## Abbreviations

A: Allelopathy
C: Resource Competition
AC: Allelopathy and Resource Competition.
