# Supplementary material for: The Impact of Competition and Allelopathy on the Trade-Off between Plant Defense and Growth in Two Contrasting Tree Species
Source: Front Plant Sci. 2016 May 4;7:594. doi: 10.3389/fpls.2016.00594 (PMC4855863; doi:10.3389/fpls.2016.00594)
Supplement: Supplementary file 1 [file Table1.docx]

**Supplementary Materials**

**Supplementary Table S1.** Concentrations of identified terpenoids (mg.g^-1^ dry weight; mean ± SE) in *Pinus halepensis* needles for the different treatments: Control; Allelopathy; Competition; Allelopathy + Competition.

|  | Control | | Allelopathy | | Competition | | Allelopathy + Competiton | |  | Control | | Allelopathy | | Competition | | Allelopathy + Competiton | |
| --- | --- | --- | --- | --- | --- | --- | --- | --- | --- | --- | --- | --- | --- | --- | --- | --- | --- |
| a-Thujene | 3.9 | ± 0.8 | 9.1 | ± 2.6 | 4.4 | ± 0.6 | 3.1 | ± 0.5 | g-Muurolene | 2.3 | ± 0.1 | 2.8 | ± 0.6 | 5.2 | ± 1.5 | 7.6 | ± 2.7 |
| a-Pinene | 13.6 | ± 2.4 | 24.6 | ± 9.4 | 224.8 | ± 110.5 | 114.0 | ± 51.8 | Germacrene D | 3.6 | ± 0.3 | 4.4 | ± 0.9 | 22.2 | ± 9.7 | 28.1 | ± 12.1 |
| Camphene | - | - | 0.2 | ± 0.1 | 3.0 | ± 1.5 | 0.7 | ± 0.5 | Phenethyl isovalerate | 1.2 | ± 0.7 | 1.5 | ± 0.9 | 4.2 | ± 3.3 | 3.4 | ± 3.2 |
| Sabinene | 4.0 | ± 0.8 | 8.0 | ± 3.8 | 5.0 | ± 1.1 | 5.8 | ± 2.7 | a-Muurolene | 31.6 | ± 4.5 | 44.3 | ± 8.2 | 77.3 | ± 17.3 | 96.9 | ± 33.7 |
| b-Pinene | 0.4 | ± 0.2 | 1.3 | ± 0.5 | 14.6 | ± 7.3 | 10.3 | ± 5.9 | Cubebol | 8.5 | ± 5.4 | 12.3 | ± 8.2 | 23.3 | ± 14.9 | 12.2 | ± 8.6 |
| b-Myrcene | - | - | 0.1 | ± 0.1 | - | - | 0.5 | ± 0.4 | d-Cadinene | 28.4 | ± 5.4 | 30.8 | ± 9.7 | 47.2 | ± 18.6 | 49.0 | ± 17.7 |
| a-Phellandrene | - | - | 0.3 | ± 0.2 | 0.1 | ± 0.1 | 0.6 | ± 0.5 | Elemol | 1.5 | ± 0.9 | 6.7 | ± 1.3 | 19.4 | ± 9.2 | 16.9 | ± 7.3 |
| Carene | 1.1 | ± 0.9 | 2.5 | ± 1.0 | 21.8 | ± 10.3 | 12.9 | ± 6.9 | Germacrene D-4-ol | 2.9 | ± 1.7 | 3.2 | ± 1.2 | 1.6 | ± 1.1 | 4.9 | ± 3.8 |
| a-Terpinene | 2.0 | ± 0.9 | 2.4 | ± 0.5 | 1.5 | ± 0.4 | 0.6 | ± 0.2 | Caryophyllene oxide | 17.9 | ± 3.5 | 29.0 | ± 7.6 | 29.5 | ± 4.7 | 36.0 | ± 9.7 |
| p-Cymene | 0.3 | ± 0.1 | 0.2 | ± 0.1 | - | - | 0.5 | ± 0.3 | Guaiol | 20.3 | ± 10.1 | 21.6 | ± 12.4 | 44.2 | ± 5.7 | 38.1 | ± 10.2 |
| Limonene | 5.8 | ± 3.4 | 8.5 | ± 2.7 | 10.1 | ± 1.4 | 12.2 | ± 3.0 | t-Muurolol | 10.2 | ± 7.0 | 5.3 | ± 3.1 | 10.1 | ± 5.5 | 6.9 | ± 4.1 |
| d-terpinene | 0.1 | ± 0.1 | 1.4 | ± 0.3 | 3.1 | ± 1.6 | 4.7 | ± 2.2 | d-Cadinol | 9.1 | ± 5.0 | 16.6 | ± 7.4 | 21.0 | ± 5.0 | 16.7 | ± 7.1 |
| Terpinen-4-ol | - | - | 0.1 | ± 0.1 | 0.5 | ± 0.3 | 0.4 | ± 0.2 | b-Eudesmol | 17.9 | ± 2.6 | 24.7 | ± 2.8 | 23.7 | ± 7.3 | 22.8 | ± 4.5 |
| Borneol acetate | 0.1 | ± 0.1 | 0.8 | ± 0.3 | 8.5 | ± 4.2 | 3.7 | ± 1.6 | a-Eudesmol | 36.6 | ± 7.1 | 52.1 | ± 16.0 | 43.2 | ± 3.9 | 51.8 | ± 13.6 |
| d-Elemene | - | - | 0.1 | ± 0.1 | 2.8 | ± 1.4 | 3.3 | ± 1.5 | b-Springene | 4.1 | ± 2.1 | 8.6 | ± 0.8 | 7.5 | ± 0.7 | 8.1 | ± 1.4 |
| a-Cubebene | 1.5 | ± 0.3 | 2.1 | ± 0.6 | 4.7 | ± 1.4 | 4.1 | ± 1.7 | Cembrene | 6.2 | ± 1.2 | 7.2 | ± 1.1 | 6.3 | ± 0.9 | 8.1 | ± 2.2 |
| Cyclosativene | 1.0 | ± 0.5 | 1.7 | ± 0.6 | 7.3 | ± 4.1 | 6.7 | ± 2.9 | Thunbergol | - | - | 2.8 | ± 2.8 | 4.8 | ± 4.8 | - | - |
| a-Ylangene | - | - | - | - | - | - | 0.1 | ± 0.1 | Sandaracopimaric acid-methyl ester | 16.4 | ± 2.8 | 21.7 | ± 5.3 | 21.1 | ± 4.3 | 17.7 | ± 2.7 |
| Copaene | 1.8 | ± 0.2 | 3.1 | ± 1.2 | 25.1 | ± 12.0 | 26.2 | ± 10.6 | Dehydroabietal | 19.1 | ± 2.1 | 27.5 | ± 5.6 | 23.4 | ± 1.7 | 65.0 | ± 20.3 |
| b-Cubebene | 1.2 | ± 0.6 | 2.3 | ± 0.6 | 3.9 | ± 1.0 | 3.8 | ± 1.3 | Isopimaric acid-methyl ester | 7.8 | ± 3.5 | 6.6 | ± 2.3 | 11.9 | ± 2.5 | 8.4 | ± 2.2 |
| b-Elemene | 0.4 | ± 0.2 | 0.4 | ± 0.3 | 3.2 | ± 1.3 | 4.1 | ± 1.7 | Levopimaric acid-methyl ester | 126.6 | ± 30.5 | 170.2 | ± 38.1 | 180.0 | ± 33.0 | 181.2 | ± 32.9 |
| b-Caryophyllene | 45.2 | ± 4.3 | 61.2 | ± 15.4 | 535.1 | ± 249.5 | 488.3 | ± 220.3 | Dehydroabietic acid-methyl ester | 103.5 | ± 19.1 | 132.1 | ± 31.9 | 100.7 | ± 9.4 | 164.0 | ± 40.4 |
| Calarene | 2.0 | ± 0.8 | 1.7 | ± 0.6 | 5.5 | ± 2.1 | 6.1 | ± 2.3 | Abietic acid_ methyl ester | 24.7 | ± 2.1 | 31.2 | ± 7.8 | 25.1 | ± 4.8 | 29.1 | ± 8.1 |
| a-Caryophyllene | 12.4 | ± 1.8 | 17.2 | ± 4.3 | 101.2 | ± 44.1 | 84.8 | ± 35.5 | Neoabietic acid-methyl ester | 133.7 | ± 16.4 | 154.8 | ± 43.0 | 156.8 | ± 33.1 | 185.4 | ± 52.7 |
| trans-b-Farnesene | 0.6 | ± 0.3 | 0.7 | ± 0.5 | 4.6 | ± 1.7 | 4.8 | ± 2.0 | Abietatrien-7-13-15-oic acid-methyl ester | 80.5 | ± 40.3 | 108.4 | ± 31.2 | 115.4 | ± 17.5 | 88.1 | ± 20.7 |
| epi-Caryophyllene | 0.7 | ± 0.5 | 0.5 | ± 0.3 | 2.8 | ± 1.1 | 1.8 | ± 0.9 | Dehydroabietic acid-15-hydroxy | 22.6 | ± 12.3 | 27.6 | ± 11.0 | 13.4 | ± 7.0 | 17.5 | ± 5.8 |
