# Supplementary material for: The Impact of Competition and Allelopathy on the Trade-Off between Plant Defense and Growth in Two Contrasting Tree Species
Source: Front Plant Sci. 2016 May 4;7:594. doi: 10.3389/fpls.2016.00594 (PMC4855863; doi:10.3389/fpls.2016.00594)
Supplement: Supplementary file 2 [file Table2.DOCX]

**Supplementary Table S2.** Concentrations of phenolic monomers and aliphatic acids (mg.g^-1^ dry weight; mean ± SE) in *Pinus halepensis needles* and *Quercus pubescens* leaves for the different treatments: Control; Allelopathy; Competition; Allelopathy + Competition.

|  | *Quercus pubescens* | | | | | | | |  | *Pinus halepensis* | | | | | | | |
| --- | --- | --- | --- | --- | --- | --- | --- | --- | --- | --- | --- | --- | --- | --- | --- | --- | --- |
|  | Control | | Allelopathy | | Competition | | Allelopathy + Competiton | |  | Control | | Allelopathy | | Competition | | Allelopathy + Competiton | |
| Azelaic acid | 1.8 | ± 1.8 | 17.1 | ± 13.4 | 1.2 | ± 1.2 | 7.5 | ± 3.0 |  | 7.4 | ± 5.5 | 4.4 | ± 1.9 | 2.8 | ± 2.1 | 4.2 | ± 2.4 |
| Malonic acid | 105.9 | ± 85.6 | 7.8 | ± 7.8 | 49.8 | ± 49.8 | - | - |  | - | - | - | - | - | - | - | - |
| Stearic acid | 202.5 | ± 106.1 | 374.5 | ± 145.9 | 290.0 | ± 135.9 | 538.7 | ± 419.4 |  | 98.3 | ± 52.9 | 25.9 | ± 19.8 | 388.6 | ± 305.5 | 58.7 | ± 56.4 |
| Citric acid | 4993.1 | ± 3773.7 | 3641.5 | ± 1591.4 | 860.5 | ± 381.9 | 3946.1 | ± 3133.6 |  | 2964.9 | ± 1230.6 | 386.7 | ± 132.2 | 3486.7 | ± 2352.0 | 1043.8 | ± 493.4 |
| Succinic acid | 55.9 | ± 28.7 | 161.2 | ± 60.1 | 237.0 | ± 79.1 | 220.4 | ± 145.6 |  | 192.4 | ± 86.4 | 146.7 | ± 99.2 | 381.1 | ± 239.5 | 72.8 | ± 25.3 |
| Palmitic acid | 11.2 | ± 5.2 | 37.6 | ± 19.4 | 26.6 | ± 9.7 | 48.5 | ± 39.5 |  | 50.6 | ± 45.6 | 1.4 | ± 0.7 | 54.0 | ± 47.6 | 3.7 | ± 2.6 |
| Lauric acid | - | - | - | - | - | - | 2.4 | ± 2.2 |  | - | - | - | - | - | - | - | - |
| 4-hydroxyacetophenone | - | - | 0.7 | ± 0.4 | - | - | - | - |  | 0.4 | ± 0.4 | 0.2 | ± 0.1 | - | - | - | - |
| Sinapic acid | 1.0 | ± 0.7 | 2.9 | ± 2.5 | 1.3 | ± 0.8 | 1.8 | ± 1.2 |  | - | - | - | - | - | - | - | - |
| Salicylic acid | 4.1 | ± 2.2 | 6.5 | ± 2.2 | 11.7 | ± 2.0 | 11.4 | ± 7.0 |  | 1.5 | ± 0.7 | 4.4 | ± 2.3 | 2.1 | ± 1.0 | 1.2 | ± 0.3 |
| 4-hydroxybenzoic acid | 1.6 | ± 0.8 | 4.1 | ± 1.6 | 4.0 | ± 0.8 | 6.7 | ± 5.1 |  | 1.4 | ± 0.5 | 1.5 | ± 0.6 | 4.5 | ± 2.0 | 1.1 | ± 0.5 |
| Syringaldehyde | 0.7 | ± 0.5 | 3.7 | ± 2.7 | 0.9 | ± 0.5 | 0.5 | ± 0.5 |  | 0.2 | ± 0.2 | - | - | 4.2 | ± 4.2 | 0.4 | ± 0.4 |
| Vanillin | - | - | 24.1 | ± 18.4 | 4.1 | ± 4.1 | 9.3 | ± 5.0 |  | 2.4 | ± 1.9 | 0.6 | ± 0.6 | - | - | 2.2 | ± 1.3 |
| Caffeic acid | 7.2 | ± 3.1 | 21.3 | ± 7.3 | 15.4 | ± 5.3 | 8.9 | ± 2.9 |  | 3.5 | ± 1.3 | 3.3 | ± 0.9 | 38.4 | ± 35.6 | 2.6 | ± 0.4 |
| Gallic acid | 4990.2 | ± 1904.9 | 3354.1 | ± 1527.2 | 3552.2 | ± 913.9 | 4128.7 | ± 1475.2 |  | 593.0 | ± 195.5 | 107.9 | ± 77.0 | 1468.3 | ± 1032.0 | 354.4 | ± 141.3 |
| Cinnamic acid | 0.4 | ± 0.4 | - | - | - | - | 0.6 | ± 0.6 |  | 0.6 | ± 0.4 | 0.5 | ± 0.3 | 1.2 | ± 0.7 | 0.1 | ± 0.1 |
| p-Coumaric acid | 19.0 | ± 8.6 | 29.8 | ± 10.7 | 20.2 | ± 3.9 | 19.2 | ± 8.1 |  | 4.7 | ± 1.3 | 5.3 | ± 2.1 | 15.5 | ± 8.9 | 6.5 | ± 2.8 |
| Benzoic acid | 15.8 | ± 9.6 | 140.6 | ± 92.4 | 34.5 | ± 17.5 | 21.6 | ± 10.8 |  | 65.8 | ± 43.4 | 6.0 | ± 3.4 | 39.0 | ± 35.9 | 6.9 | ± 6.9 |
| Gentisic acid | 245.8 | ± 204.0 | 162.6 | ± 112.1 | 116.6 | ± 76.2 | 7.7 | ± 7.7 |  | 256.0 | ± 228.5 | 2.5 | ± 1.8 | 17.3 | ± 17.3 | 21.0 | ± 21.0 |
| Vanillic acid | 12.7 | ± 6.4 | 22.5 | ± 10.1 | 8.5 | ± 3.9 | 18.6 | ± 8.2 |  | 19.7 | ± 6.6 | 15.5 | ± 7.3 | 15.4 | ± 10.4 | 62.1 | ± 48.1 |
| Benzaldehyde | 0.1 | ± 0.1 | 6.6 | ± 5.7 | 0.2 | ± 0.1 | 0.6 | ± 0.3 |  | 2.4 | ± 0.9 | 0.4 | ± 0.2 | 3.7 | ± 2.1 | 0.4 | ± 0.4 |
| Acetophenone | 1.9 | ± 1.9 | 17.2 | ± 9.1 | 4.0 | ± 2.6 | 1.3 | ± 1.3 |  | 5.5 | ± 3.5 | - | - | 10.8 | ± 5.5 | 0.7 | ± 0.7 |
