# Supplementary material for: The Impact of Competition and Allelopathy on the Trade-Off between Plant Defense and Growth in Two Contrasting Tree Species
Source: Front Plant Sci. 2016 May 4;7:594. doi: 10.3389/fpls.2016.00594 (PMC4855863; doi:10.3389/fpls.2016.00594)
Supplement: Supplementary file 3 [file Table3.DOCX]

**Supplementary Table S3.** Results of SIMPER analysis showing the ten most important phenolics and terpenoids for which variations contribute (Contribution in %) to dissimilarities between control and each treatment. Below, the “S” indice showing the compounds for which variations are mostly specific to A (close to 1) or C (close to -1).

| **Treatment** | ***Quercus* *pubescens* Phenolics** | | ***Pinus halepensis* phenolics** | | ***Pinus halepensis* terpenoids** | |
| --- | --- | --- | --- | --- | --- | --- |
|  | **Compounds** | **Contribution (%)** | **Compounds** | **Contribution (%)** | **Compounds** | **Contribution (%)** |
| **Allelopathy** | 4-hydroxyacetophenone | 7.564 | Gentisic acid | 10.67 | a-Thujene | 8.852 |
|  | Benzoic acid | 7.378 | Salycilic acid | 10.43 | b_Springene | 7.708 |
|  | Vanillin | 7.364 | 4-hydroxyacetophenone | 10.02 | Abietatrien-7-13-15-oic acid- methyl ester | 6.166 |
|  | Acetophenone | 7.277 | Vanillin | 9.714 | Dehydroabietic acid-15-hydroxy_ | 5.842 |
|  | Benzaldehyde | 7.203 | Benzoic acid | 9.313 | Sandaracopimaric acid- methyl ester | 5.658 |
|  | Azelaic acid | 6.993 | Azelaic acid | 8.9 | Sabinene | 4.772 |
|  | Syringaldehyde | 6.721 | Palmitic acid | 7.266 | a-Terpinene | 4.701 |
|  | Caffeic acid | 6.435 | Vanillic acid | 6.282 | t-Muurolol | 4.453 |
|  | Malonic acid | 5.83 | Citric acid | 5.912 | Levopimaric acid- methyl ester | 4.081 |
|  | Sinapic acid | 5.467 | Acetophenone | 4.762 | Isopimaric acid-methyl ester | 3.997 |
| **Competition** | Malonic acid | 14.4 | Caffeic acid | 7.188 | Camphene | 6.286 |
|  | Gentisic acid | 12.64 | Syringaldehyde | 7.132 | Borneol acetate | 5.8 |
|  | Citric acid | 12.38 | 4-hydroxybenzoic acid | 7.034 | a-Pinene | 4.943 |
|  | Gallic acid | 9.536 | p-Coumaric acid | 7.005 | Thunbergol | 4.222 |
|  | Succinic acid | 9.493 | Stearic acid | 6.549 | Carene | 3.756 |
|  | Salycilic acid | 7.608 | Gallic acid | 5.87 | Elemol | 3.006 |
|  | Caffeic acid | 7.447 | Vanillin | 5.518 | epi-Caryophyllene | 2.947 |
|  | Cinnamic acid | 5.995 | 4-hydroxyacetophenone | 5.476 | b-Pinene | 2.938 |
|  | p-Coumaric acid | 4.891 | Gentisic acid | 5.388 | a-Caryophyllene | 2.881 |
|  | Vanillic acid | 3.897 | Succinic acid | 5.214 | Cyclosativene | 2.701 |
| **Allelopathy + Competition** | Lauric acid | 9.506 | Vanillic acid | 68.65 | b-Springene | 3.089 |
|  | 4-hydroxybenzoic acid | 9.416 | 4-hydroxyacetophenone | 3.993 | Dehydroabietal | 2.662 |
|  | Malonic acid | 8.33 | Vanillin | 3.948 | a-Terpinene | 2.659 |
|  | Salycilic acid | 8.289 | Gentisic acid | 3.909 | d-terpinene | 2.595 |
|  | Stearic acid | 8.161 | Azelaic acid | 3.603 | Limonene | 2.544 |
|  | Palmitic acid | 8.084 | Benzoic acid | 3.538 | a-Muurolene | 2.54 |
|  | Gentisic acid | 8.075 | Palmitic acid | 2.696 | g-Muurolene | 2.485 |
|  | Succinic acid | 7.314 | Citric acid | 1.79 | b-Cubebene | 2.416 |
|  | Cinnamic acid | 7.14 | Benzaldehyde | 1.736 | b-Elemene | 2.385 |
|  | Citric acid | 6.762 | Acetophenone | 1.659 | Germacrene D | 2.357 |
|  | **Compounds** | **S** | **Compounds** | **S** | **Compounds** | **S** |
|  | 4-hydroxyacetophenone | 1 | Salycilic acid | 0.944 | a-Thujene | 0.887 |
|  | Benzoic acid | 0.762 | Vanillic acid | 0.556 | Sabinene | 0.830 |
|  | Benzaldehyde | 0.714 | Benzoic acid | 0.5 | a-Eudesmol | 0.679 |
|  | Vanillin | 0.667 | Citric acid | 0.444 | Abietic acid_methyl ester | 0.566 |
|  | Azelaic acid | 0.619 | Gentisic acid | 0.444 | Dehydroabietic acid_methyl ester | 0.566 |
|  | Acetophenone | 0.524 | Palmitic acid | 0.333 | Sandaracopimaric acid-methyl ester | 0.453 |
|  | Syringaldehyde | 0.429 | 4-hydroxyacetophenone | 0.278 | Caryophyllene oxide | 0.434 |
|  | Sinapic acid | 0.095 | Acetophenone | 0.278 | a-Phellandrene | 0.396 |
|  | Caffeic acid | -0.048 | Azelaic acid | 0.278 | Neoabietic acid-methyl ester | 0.396 |
|  | Lauric acid | -0.048 | Benzaldehyde | 0.222 | b-Myrcene | 0.377 |
|  | p-Coumaric acid | -0.095 | Vanillin | 0.167 | Cembrene | 0.358 |
|  | Palmitic acid | -0.143 | Cinnamic acid | 0.056 | Dehydroabietic acid-15-hydroxy_ | 0.358 |
|  | Vanillic acid | -0.190 | Succinic acid | -0.167 | Germacrene D-4-ol | 0.358 |
|  | 4-hydroxybenzoic acid | -0.333 | Gallic acid | -0.5 | Abietatrien-7-13-15-oic acid-methyl | 0.340 |
|  | Cinnamic acid | -0.381 | 4-hydroxybenzoic acid | -0.611 | Levopimaric acid-methyl ester | 0.340 |
|  | Malonic acid | -0.381 | p-Coumaric acid | -0.667 | Limonene | 0.340 |
|  | Stearic acid | -0.381 | Stearic acid | -0.667 | d-Cadinol | 0.321 |
|  | Gallic acid | -0.429 | Caffeic acid | -0.944 | Inconnu -1026_ | 0.321 |
|  | Gentisic acid | -0.476 | Syringaldehyde | -0.944 | a-Terpinene | 0.283 |
|  | Citric acid | -0.571 |  |  | Dehydroabietal | 0.283 |
|  | Succinic acid | -0.619 |  |  | p-Cymene | 0.2641 |
|  | Salycilic acid | -0.714 |  |  | d-Cadinene | 0.189 |
|  |  |  |  |  | b-Springene | 0.170 |
|  |  |  |  |  | Guaiol | 0.170 |
|  |  |  |  |  | t-Muurolol | 0.170 |
|  |  |  |  |  | Isopimaric acid-methyl ester | 0.038 |
|  |  |  |  |  | a-Ylangene | 0 |
|  |  |  |  |  | b-Eudesmol | 0 |
|  |  |  |  |  | d-terpinene | 0 |
|  |  |  |  |  | a-Muurolene | -0.019 |
|  |  |  |  |  | g-Muurolene | -0.019 |
|  |  |  |  |  | b-Cubebene | -0.075 |
|  |  |  |  |  | Calarene | -0.075 |
|  |  |  |  |  | Phenethyl isovalerate | -0.075 |
|  |  |  |  |  | a-Cubebene | -0.132 |
|  |  |  |  |  | Cubebol | -0.132 |
|  |  |  |  |  | Thunbergol | -0.151 |
|  |  |  |  |  | unknown-58 | -0.226 |
|  |  |  |  |  | b-Elemene | -0.226 |
|  |  |  |  |  | Germacrene D | -0.283 |
|  |  |  |  |  | Terpinen-4-ol | -0.283 |
|  |  |  |  |  | trans-b-Farnesene | -0.283 |
|  |  |  |  |  | epi-Caryophyllene | -0.378 |
|  |  |  |  |  | d-Elemene | -0.472 |
|  |  |  |  |  | Elemol | -0.528 |
|  |  |  |  |  | Copaene | -0.547 |
|  |  |  |  |  | Cyclosativene | -0.566 |
|  |  |  |  |  | b-Caryophyllene | -0.698 |
|  |  |  |  |  | Carene | -0.736 |
|  |  |  |  |  | a-Caryophyllene | -0.736 |
|  |  |  |  |  | Borneol acetate | -0.774 |
|  |  |  |  |  | b-Pinene | -0.792 |
|  |  |  |  |  | a-Pinene | -0.811 |
|  |  |  |  |  | Camphene | -0.8680 |
