# Supplementary figures and images for: The Impact of Competition and Allelopathy on the Trade-Off between Plant Defense and Growth in Two Contrasting Tree Species
Source: Front Plant Sci. 2016 May 4;7:594. doi: 10.3389/fpls.2016.00594 (PMC4855863; doi:10.3389/fpls.2016.00594)

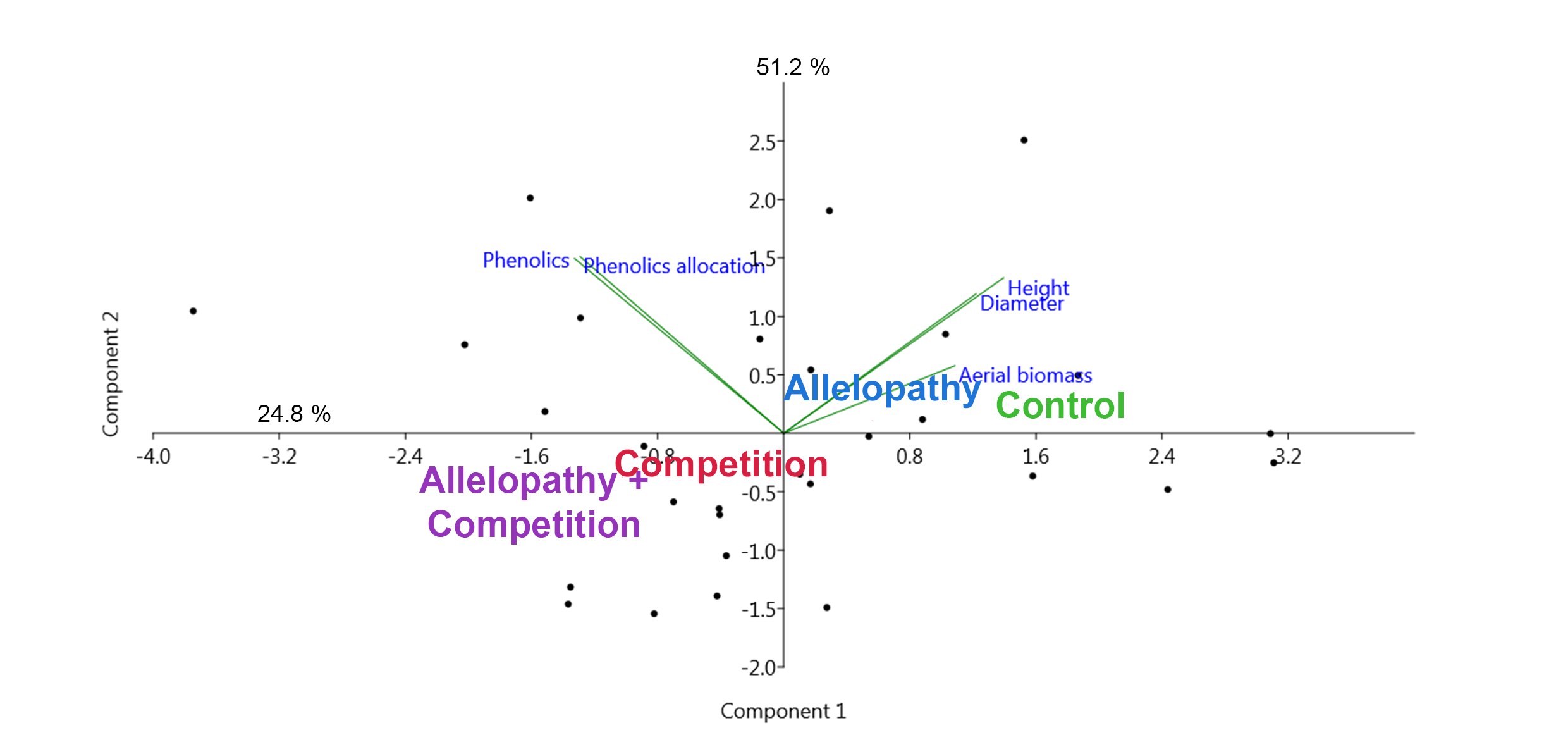

Supplement: Supplementary file 4 [file Image1.TIF]
